# The impact of ethnicity information in single best answer questions on student performance, item response time and psychometric properties

**DOI:** 10.1007/s10459-025-10489-6

**Published:** 2025-12-05

**Authors:** Jenanan Mohan, Michaela Pishia, Duaa Alim, Patrick J. McGown, Anjali Amin, Richard J. Pinder, Omid Halse, Celia Brown, Amir H. Sam

**Affiliations:** Imperial College School of Medicine, Imperial College London, London, UK

**Keywords:** Ethnicity, Race, Assessment, Undergraduate medical education, Single best answer questions

## Abstract

Inclusion of patient age and sex in single best answer (SBA) questions is standard practice, whereas ethnicity is typically included only when considered clinically relevant. This selective use may inadvertently cue students or introduce bias. This study examined whether including patient ethnicity in SBA questions influences student performance, response time and item psychometric properties. A prospective randomised controlled study was conducted using two versions of a final-year formative assessment. In each version, approximately half of the questions included the patient’s ethnicity, while the corresponding questions in the alternate version omitted this information. Students were randomly assigned to one of the two exam versions. Two hundred and sixty-seven final-year medical students participated in the study. The mean score for questions without ethnicity was 80.3% and for questions with ethnicity was 79.8% (mean difference = −0.5 pp, *p* = 0.12). On average, students spent 1.6 seconds longer to answer questions with ethnicity compared to questions without ethnicity (*p* < 0.01). In SBA questions with and without ethnicity, there was no significant difference in mean facility (79.8% vs. 80.3%, *p* = 0.17) or mean item–total score point-biserial correlation (0.20 vs. 0.21, *p* = 0.63). These indicate that including patient ethnicity did not significantly impact overall student performance or mean item-level psychometric properties. However, it was associated with longer response times, suggesting a small but significant increase in cognitive load. Educators involved in assessment design should carefully consider this added cognitive burden when deciding whether to include patient ethnicity in future assessments.

## Introduction

Addressing racial inequalities is essential in creating a fairer future for both patients and healthcare professionals (British Medical Association, 2021). Many individuals from racial and ethnic minority backgrounds face distinct barriers within the healthcare system, including limited access to high-quality services and biases in clinical decision-making (Sarto et al., 2013). Although the inequalities observed across different ethnic groups are complex and involve numerous interrelated factors (The King’s Fund, 2023), recent data suggest that patients from non-white communities are more likely to be misdiagnosed (Newman-Toker et al., 2022). As a result, it is increasingly important for the medical profession to address the racial and ethnic biases that contribute to these disparities.

In the United Kingdom (UK), the government uses the term ethnicity rather than race when referring to people’s backgrounds (UK Government, 2024). While some countries still categorise populations according to race, ethnicity in the UK is viewed as a more positive and inclusive identity, reflecting shared cultural heritage, language, and religious practices (The Law Society, 2025). Consequently, diversity questionnaires in the UK, such as the Census, routinely ask about ethnicity rather than race (Office for National Statistics, 2022). Patients’ ethnic backgrounds can influence clinical decision-making, sometimes in subtle ways. Research indicates that healthcare providers, including medical students, may hold implicit biases that affect their perceptions and decisions. For instance, a qualitative study in Australia and Hawaii found that final-year students exhibited “subtle biases” in how they discussed and anticipated caring for an Indigenous patient when the patient’s ethnicity was explicitly stated (Ewen et al., 2015). In New Zealand, the Bias and Decision Making in Medicine (BDMM) study showed final-year students had implicit positive biases toward New Zealanders of European descent and tended to view Māori (Indigenous) patients as less compliant with treatment recommendations (Cormack et al., 2018). These biases were present even if overt prejudice was minimal, suggesting that unconscious stereotypes influenced clinical judgment.

Likewise, a United States study revealed that a significant number of medical trainees endorsed false biological beliefs about racial differences (e.g. believing Black patients have higher pain tolerance), which correlated with undertreating pain in Black patients (Hoffman et al., 2016). In that study, white medical students and resident doctors who held such false beliefs rated Black patients’ pain lower and made less appropriate treatment decisions. These examples illustrate that a patient’s ethnicity and the provider’s preconceptions about it may impact diagnostic reasoning, expectations of patient behaviour, and management decisions in real-life clinical scenarios. Such biases contribute to healthcare disparities and underscore why medical training must address race and ethnicity to ensure that future doctors make decisions based on individual clinical need rather than stereotypes.

The importance of preparing students to care for patients from a variety of backgrounds is widely accepted within medical education (Medical Schools Council, 2021). Inclusive and diverse learning environments have been shown to lead to better educational outcomes for medical trainees and more compassionate, higher-quality care for patients (Medical Schools Council, 2021). However, in traditional curricula and assessments, patient demographics beyond age and sex are often overlooked. An intersectional approach, which considers how factors such as socio-economic status, ethnicity, language, and other identity characteristics interact to influence patient care, is also rarely represented in assessment materials. For instance, most undergraduate single best answer (SBA) exam questions provide only age and sex, and do not represent the breadth of patient backgrounds encountered in actual practice (Shepherd et al., 2024). This lack of diversity in assessment materials can contribute to a “hidden curriculum”, implying a culturally uniform patient population and unintentionally fostering unconscious biases (Lee et al., 2022). Cultivating an understanding of cultural safety among medical students is an effective pathway to advancing healthcare equity (Curtis et al., 2019).

In the UK, neither the General Medical Council (GMC) nor the Medical Schools Council Assessment Alliance (MSCAA) explicitly list ethnicity as a required element in written exam questions. The traditional ‘house style’ for SBA questions is to provide the patient’s age, sex, and other details relevant to the case (such as symptoms and key history), while omitting irrelevant information (Medical Schools Council, 2018). The GMC’s broad standards emphasise fairness in assessment, however, these do not mandate specific content such as ethnicity descriptors in questions (General Medical Council, 2015, 2011).

Recent research confirms that patient ethnicity is rarely included in written exam questions, especially compared to age and sex. In an audit of 3,566 medical school exam questions across three schools, one in Australia and two in Europe, only 7.2% of questions that presented a patient mentioned the patient’s ethnicity (Marjadi, 2024). By contrast, 89% of such questions specified the patient’s age and 94% specified the patient’s sex. Other identity characteristics were even less frequently portrayed (e.g. sexual orientation in 1.1% of cases). These findings highlight a stark under-representation of cultural diversity in assessment content. A similar pattern has been observed in the United States: an analysis of a US Medical Licensing Examination (USMLE) Step 1 question bank found that non-white ethnic groups were under-represented in question vignettes and appeared in a narrower range of disease contexts (Ripp & Braun, 2017). In that study, when race was mentioned, it was typically central to the diagnosis (e.g. sickle cell disease in a patient of African descent), whereas white patients were often treated as the default with ethnicity omitted (Krishnan et al., 2019). Taken together, the literature indicates that while age and sex are almost always given in exam scenarios, ethnicity is included far less often.

The body of research specifically examining the inclusion of ethnicity in medical exam questions is relatively small and has been described as an under-researched area (Marjadi, 2024). Most of the literature on diversity in medical education has focused on curriculum content, clinical teaching, or differential student performance (for example, the awarding gap between ethnic minority and white medical students), rather than analysing assessment items themselves (Muntinga et al., 2015; Dogra et al., 2009; Brown et al., 2023). Only in the last decade have scholars begun to scrutinise how demographic factors, including ethnicity, are represented in exam materials. Key studies have emerged in the past few years, for instance, Ripp and Braun’s analysis of a U.S. question bank (Ripp & Braun, 2017) and the multi-school audit by Marjadi et al. (Marjadi, 2024), but data from UK medical school assessments specifically are still limited.

There is a significant lack of research on how the inclusion of ethnicity in medical written examination questions affects candidates’ responses. One study indicates that mentioning a patient’s race or ethnicity can act as a cue (Arnason et al., 2023). A content analysis of U.S. question banks found that race was often used either as a deliberate “clue”, such as specifying a patient is black to hint at hypertension-related complications, or as a “distraction” that steered students toward a stereotypical but incorrect diagnosis (Cerdeña et al., 2021). However, to the best of our knowledge, no prospective studies have examined the psychometric and cognitive effects of including patient ethnicity in undergraduate written assessment items. To address this gap, we posed the following research question:

- Does the inclusion of patient ethnicity in written medical exam questions affect medical student performance, response time and item-level psychometric properties (question facility and discrimination)?

## Methods

### Study design

This study used a randomised, prospective parallel-forms (split-half) design. Two versions of the same SBA question examination (Paper A and Paper B) were created, with each SBA question in the ‘pick one from five’ format. In Paper A, odd-numbered questions included the patient’s ethnicity, whereas in Paper B, even-numbered questions included ethnicity.

### Participants and setting

The study was conducted at Imperial College London, UK. Eligible participants were students enrolled in their final year of medical school. All eligible students were notified of a formative written assessment, which would be similar in design to their summative written assessment, that would take place over two days. Data collection took place in January 2025. All students in their final year (*n* = 339) were randomly divided into two groups, one assigned Paper A and the other assigned Paper B.

To minimise bias, students were informed that the study aimed to explore formative exam processes and identify areas where they might need additional support before their summative assessment. Before the assessment began, all students were asked to indicate whether they consented to their data being analysed. Students were permitted to complete the formative assessment regardless of whether or not they had given consent, and participation in the formative assessment was not mandatory. Students were encouraged to complete the assessment as it provided an authentic practice opportunity ahead of their summative assessment in the following weeks. Participants were not informed that the research also aimed to evaluate the impact of including ethnicity in written examination questions, as awareness of this objective could have introduced bias into their responses to the SBA items.

Both Paper A and Paper B contained 200 single best answer (SBA) items, matching the length of the summative final examination. In the summative format, the assessment is administered over two consecutive days, with students completing 100 SBA items on each day. This approach was replicated in the formative assessment used in this study to mirror the summative assessment experience (Medical Schools Council, 2024). Students were allocated two hours per section, giving them a total of four hours to complete all 200 SBA items over the two days. Students entitled to reasonable adjustments, such as additional time or scheduled breaks, received these accommodations. All students sat the formative written papers remotely via the online exam platform risr/assess 8.14 (Fry-IT, London, UK). Due to the remote nature of the assessment, no invigilation was in place. Students were instructed to approach the examination under summative conditions to maximise its educational value.

### Examination design and variables

Two versions of the same SBA examination – Paper A and Paper B – were developed. As this was the first year that final-year medical students would be taking the newly introduced UK Medical School Applied Knowledge Test (MS AKT), the formative examination was designed using MS AKT practice papers provided by the Medical Schools Council (Medical Schools Council, 2024). Each version of the paper comprised approximately 50% of questions that included ethnicity and 50% that omitted it. In Paper A, odd-numbered questions included the patient’s ethnicity, while the even-numbered questions did not; in Paper B, this pattern was reversed. This design allowed each ‘ethnicity’ question to be paired with a corresponding ‘non-ethnicity’ question for data analysis (Appendix 1).

An example is shown below:

1. Without ethnicity – A 62 year old man has 2 months of increasing shortness of breath and chest pain.
2. With ethnicity – A 62 year old Indian man has 2 months of increasing shortness of breath and chest pain.

The exam platform randomised the order of the questions within each paper for each student so that no two students received the same sequence. This approach aimed to minimise potential bias arising from students anticipating patterns in ethnicity inclusion.

To make the examinations as authentic as possible, the demographic composition of London according to the 2021 Census was used when assigning ethnicity to questions (Office for National Statistics, 2022). This provided guidance on how many questions should represent each ethnic group, as summarised in Table 1. For the category ‘any other ethnic background’, we drew on the more granular data from the Office for National Statistics to identify the largest represented groups (Office for National Statistics, 2022). Ethnicities were then randomly assigned to questions to reduce the risk of bias and minimise the introduction of ‘cultural clues’ (Cerdeña et al., 2021).

**Table 1:** Number of questions in which each ethnicity was included in Paper A and Paper B (each paper contained 200 questions)

Ethnicity	Number of Questions (Paper A)	Number of Questions (Paper B)
White British	35	35
White Irish	2	2
Any Other White Background	13	13
Bangladeshi	4	4
Chinese	2	2
Indian	8	8
Pakistani	4	4
Any Other Asian Background	4	4
Black African	8	7
Black Caribbean	4	4
Any Other Black Background	2	2
Mixed White and Asian	2	2
Mixed White and Black Caribbean	2	2
Any Other Mixed/Multiple Ethnic Background	2	2
Arab	2	2
Any Other Ethnic Background	4	4
Total	98	97

Following ethnicity allocation, all questions were reviewed by four UK-based physicians with at least three years of clinical experience to ensure that adding ethnicity details did not alter the correct answers. Whenever a question’s answer changed after ethnicity was added, the randomisation process was repeated with a different ethnicity until the correct answer remained consistent. In a few instances, for example ‘research methods’ questions, adding ethnicity was considered unsuitable. After review, five of the 200 questions were deemed inappropriate for the inclusion of ethnicity and were excluded from analysis. After this process, all the questions were reviewed by two assessment leads.

### Statistical methods

Statistical analysis was performed using GraphPad Prism 10.4.1 (GraphPad Software, Boston, Massachusetts, USA). Cronbach’s alpha was calculated for both Paper A and Paper B to assess the internal consistency and reliability of each exam version. A statistical comparison of the Cronbach’s alpha coefficients was conducted to determine whether there was a significant difference between the two values (Diedenhofen & Musch, 2016). This analysis provided evidence that both papers were equally effective in measuring a single underlying construct, despite differences in which questions included ethnicity.

### Student performance

Each student answered 200 questions and scores are expressed as percentages. For each student, the difference between their percentage scores on questions without ethnicity and with ethnicity was calculated, treating these as paired data. The mean and standard deviation of these differences were then determined. A paired t-test was performed to evaluate the significance of the difference in percentage scores.

### Response time

The exam platform, risr/assess 8.14, provided a breakdown of the time each student spent on each question in seconds. The mean time taken per question was calculated for each group of students who completed Paper A and Paper B. The difference in mean time between questions that included ethnicity and those that did not was then determined. A paired t-test was performed to assess whether this difference was statistically significant.

### Question facility

Question facility is defined as the proportion of candidates who correctly answer a given test item (McCrossan et al., 2022). The facility of each question was calculated, and the difference in facility between questions that included ethnicity and those that did not was analysed using a paired t-test. Because including ethnicity has the potential to either simplify or complicate a question, its effect on item facility could vary in direction. To account for this, the absolute difference in facility between the two versions of each item was also calculated. For each question pair, the version with the higher facility, whether it included ethnicity or not, was compared to the version with the lower facility. As the differences were positively skewed, a Wilcoxon matched-pairs signed-rank test was used to determine the statistical significance of this absolute difference.

### Question discrimination

Question discrimination was assessed using the point-biserial correlation coefficient between performance on each individual item and the total score (McCrossan et al., 2022). A paired t-test was then conducted to determine whether the difference in point-biserial correlation coefficients between the two groups of questions was statistically significant.

### Study size

An a priori power analysis using G*Power 3.1.9.6 for Mac OS X (Department of Psychology at Heinrich Heine University Düsseldorf, Germany) indicated that a minimum sample size of 199 participants was required to detect a small effect size of 0.2 standard deviations, with 80% power and an alpha level of 0.05, when assessing whether adding ethnicity to written examination questions influenced medical students’ performance. This calculation was performed using a paired t-test, assuming an expected standard deviation of 10 percentage points (pp), based on similar written examinations. Given the expected sample size, this study was highly likely to be appropriately powered to detect even small differences associated with the inclusion of ethnicity in exam questions.

### Ethical approval

This study was conducted in accordance with the British Educational Research Association (BERA) ethical guidelines for educational research and received approval from the Education Ethics Review Process (EERP) at Imperial College London (Reference ID: EERP2425-050). Informed consent was obtained from all the participants.

## Results

A total of 339 final-year students were eligible to participate in the study. Of these, 18 students did not attend the assessment, 20 did not consent to have their data analysed, and 34 completed only the first set of 100 questions and did not attend the second day of the formative examination. This resulted in a final sample of 267 students: corresponding to a participation rate of 78.8%. To verify that both groups were academically comparable, the mean of the students’ most recent written summative examination scores, as provided by the medical school, was calculated. This showed that Group A had a mean score of 72.4%, and Group B had a mean score of 73.3%. Cronbach alphas for Paper A and Paper B were both 0.91 (*p* = 0.70), indicating no statistically significant difference in internal consistency between the two versions of the written examination (Diedenhofen & Musch, 2016).

### Student performance

**Fig. 1:**
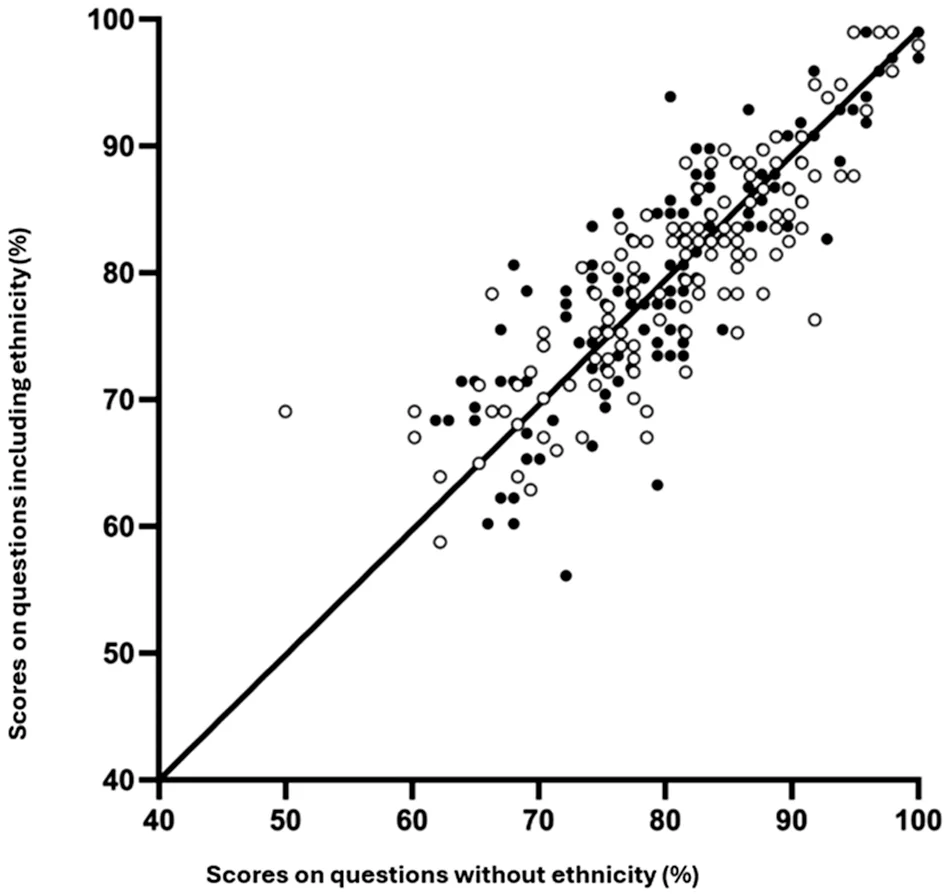
A scatter diagram of individual students’ percentage scores on SBA questions with and without ethnicity. The 45-degree line shows where performance on the two sets of questions is equal. The black coloured data set indicates students who completed Paper (**A**) examination written questions, and the white coloured data set indicates students who completed Paper (**B**) examination written questions

Students scored a mean of 0.5 pp lower on questions that included ethnicity compared to those that did not, but this difference was not statistically significant (*t* = 1.56, *p* = 0.12), as shown in Table 2. A scatter diagram of students’ scores on questions with and without ethnicity is shown in Fig. 1, with the points clustered symmetrically around the 45-degree line.

**Table 2:** Mean percentage score, mean time per question, mean question facility, and mean item–total point-biserial correlation. SD = standard deviation

Summary Statistics	Questions With Ethnicity	Questions Without Ethnicity	Paired t	*p*
**n = 267 students**				
**Mean percentage score (SD)**	79.8 (8.7)	80.3 (8.8)	1.56	0.12
**Mean time per question in seconds (SD)**	55.8 (23.8)	54.2 (23.4)	3.95	< 0.01
**n = 195 questions**				
**Mean question facility (%) (SD)**	79.8 (16.7)	80.3 (16.1)	1.39	0.17
**Mean item–total score point-biserial correlation (SD)**	0.20 (0.11)	0.21 (0.12)	0.48	0.63

### Response time

Students spent a mean of 1.6 additional seconds (standard deviation = 5.6 seconds) on questions that included ethnicity compared to those that did not. This difference was statistically significant (*t* = 3.95, *p* < 0.01).

### Question facility

Of the 195 analysed items, 98 items demonstrated a decrease in facility when ethnicity was included, while 97 items demonstrated an increase in facility. Questions with ethnicity had a mean difference of −0.5 pp (standard deviation = 4.86) compared to questions without ethnicity, which was not statistically significant (*p* = 0.17). However, acknowledging that adding ethnicity might increase or decrease a question’s difficulty, the median absolute difference in facility was 3.1 pp, a statistically significant finding (W = 19110, *p* < 0.01).

### Question discrimination

Adding ethnicity to the questions did not result in a statistically significant difference in item discrimination (mean item–total score point-biserial correlation = 0.20 for questions with ethnicity vs. 0.21 for those without; *t* = 0.48, *p* = 0.63).

## Discussion

We examined whether including patient ethnicity in SBA questions influenced student performance, item response time, difficulty and discrimination. The analysis indicated that student performance was minimally impacted by the inclusion of ethnicity in assessment items. Students scored a mean of only 0.5 pp lower on questions containing ethnicity compared to identical questions without ethnicity. This small difference was not statistically significant, suggesting that the ethnicity component alone may not influence overall student outcomes. However, it is important to consider that ethnicity content might influence question difficulty differently across items. The inclusion of ethnicity-related details may have rendered some items more straightforward, for instance by providing contextual clues (Cerdeña et al., 2021), whereas in others it may have introduced additional complexity by necessitating consideration of other variables. Including ethnicity reduced facility in about half of the items and increased it in the others. This balanced pattern suggests a neutralising effect, with opposing shifts in difficulty leading to little overall change in student performance. These findings suggest that incorporating ethnicity into SBA questions does not significantly affect student performance overall. Nevertheless, it remains important to carefully consider how ethnicity-related content may influence item difficulty in individual questions.

The results indicated that students spent a mean of 1.6 seconds extra per question when ethnicity was mentioned. For a 200-item exam like the MS AKT (Medical Schools Council, 2023), this could translate into an additional 5–6 minutes overall. The average word count of ethnicity questions was 1.7 words longer compared to non-ethnicity questions. Adding a single extra word should only require about 0.2–0.3 seconds (Carver, 1990), implying that the longer response times involve more than simply reading extra words. One possible explanation is that introducing ethnicity may increase cognitive burden. Cognitive burden (or load) refers to the mental effort required by a task, which can vary based on the task’s inherent complexity (Noroozi & Karami, 2022). Any additional details may prompt a shift towards Type 2 thinking, involving more deliberate and analytical reasoning processes. The literature has examined how various item characteristics—such as vignette complexity and linguistic difficulty—can influence student performance (Hernandez et al., 2021; Zhang et al., 2015). However, to the best of our knowledge, previous research has not investigated whether specifying a patient’s ethnicity in medical written exams adds to students’ cognitive load. Consequently, if all exam questions were to include ethnicity, the potential increase in time required per question would need to be taken into account

The results show that the facility difference between identical questions with and without ethnicity had a mean of −0.5 pp. However, since adding ethnicity may influence questions in different directions, it is important to also consider the median absolute difference in facility, which was 3.1 pp, a statistically significant finding. For instance, a question on the management of hypertension had a facility variation of 11 pp with students performing better when ethnicity was included. Hypertension is a diagnosis in which ethnicity – particularly within black populations – has relevance in the management pathway. Both international and UK guidelines recommend different first-line pharmacological therapy for Black patients compared to other ethnicities (Unger et al., 2020; National Institute for Health and Care Excellence, 2023). It can be theorised that by explicitly stating ethnicity in the stem, thereby removing uncertainty, the question was made easier.

In contrast, in another question describing a gentleman with shortness of breath, haemoptysis, and pleural effusion, the addition of Indian ethnicity resulted in a 9.8 pp decrease in correct student responses compared with the version without ethnicity. The performance difference suggests that students were influenced by the explicit mention of ethnicity and potentially made premature assumptions about geographically associated diseases such as tuberculosis, despite clinical indicators supporting an alternative diagnosis. The ethnicity cue may have prompted students to disproportionately consider stereotypically associated conditions, reflecting cognitive biases such as premature diagnostic closure. This finding underscores the unintended consequences that inclusion of ethnicity, both when clinically relevant and irrelevant, can have on student reasoning and diagnostic accuracy. It is possible that the traditional use of ethnicity in assessments purely as a cue towards a diagnosis may have encouraged automaticity in answering, thereby further reinforcing stereotypes and biases. At the same time, while some may argue that the inclusion of ethnicity could introduce unnecessary complexity, it is important to acknowledge that ethnicity is a reality of clinical practice and can influence decision-making in certain contexts. This underscores the need for thoughtful integration of ethnicity in assessment design, ensuring it is used in a way that reflects its genuine clinical relevance while minimising the risk of reinforcing unconscious biases.

The inclusion of ethnicity did not lead to any significant change in item discrimination. Item discrimination reflects an item’s ability to differentiate between higher-performing and lower-performing students, typically capturing whether stronger candidates consistently outperform weaker candidates on that specific question. One possible explanation for the non-significant change in discrimination is that ethnicity may not serve as a selective clue for students who already possess the relevant clinical knowledge. In other words, regardless of whether ethnicity was mentioned or not, students with a solid grasp of the underlying concept likely arrived at the correct answer, whereas less prepared students continued to find the item difficult. Additionally, for questions focused on core medical concepts rather than demographic details, the inclusion of an ethnic descriptor may not have been substantial enough to meaningfully distinguish top performers from lower performers. Well-written questions that rely on core principles, rather than superficial clues, might naturally maintain their discriminatory power (Walsh et al., 2016). Therefore, although students spent slightly more time on questions that included ethnicity, this did not translate into differences in how effectively the questions discriminated between varying levels of student ability.

There were several limitations to this study. First, the questions were administered in a formative rather than a summative exam, so students might not have approached them with the same level of seriousness as they would in a high-stakes setting. Second, the practice questions used are publicly available on the Medical Schools Council website for the MS AKT, which could bias the results if students had already accessed this material. We did not have data on prior use of the open question bank, so the extent of this potential bias is unknown. There may also be a response bias given that not all eligible students participated, which could have potentially influenced the study’s findings.

Data on participant ethnicity were not collected in our study. Future research with larger sample sizes should address this limitation to allow examination of potential performance differences among students from different ethnic backgrounds. Another limitation is that the study did not undertake a detailed thematic analysis of question types or topics. Such an analysis could help identify whether particular areas of clinical practice or applied knowledge are more likely to be influenced by the inclusion of ethnicity. This should be explored in future research to better understand any underlying patterns in how ethnicity affects question difficulty. Finally, although participants were asked to complete the online exam under exam-like conditions, there was no practical way to be sure they refrained from using external resources as there was no invigilation during the assessment.

Ethnicity remains an authentic aspect of clinical practice. While incorporating ethnicity into written examination questions may help foster diversity and better reflect the demographics of real-world patient populations, this approach must be balanced against potential challenges such as the added cognitive burden and the extra time required for completion. Future studies should examine in greater detail the impact of including ethnicity information on the best answer and the distractors in SBA questions. Think-aloud studies could provide insight into how students perceive and process questions that include ethnicity, informing best practices for incorporating demographic details without compromising exam performance or fairness.

## Data Availability

The data supporting the findings of this study are not openly available due to sensitivity considerations but can be obtained from the corresponding author upon reasonable request. The data are stored in controlled-access repositories at Imperial College London.

## Appendix 1

### Example question without ethnicity

A 62 year old man has 2 months of increasing shortness of breath and chest pain. He is now unable to lie flat. For the past 2 weeks, he has also had a productive cough which was flecked with blood on two occasions. He had a myocardial infarction 6 months ago, at which point he stopped smoking. His temperature is 37.1 °C, BP is 126/66 mmHg, respiratory rate is 24 breaths per minute, and oxygen saturation is 93% breathing air.

Investigations:

Chest X-ray:

moderate right sided pleural effusion

Pleural aspirate protein content 56 g/L

Which is the most likely underlying diagnosis?

A. Bacterial pneumonia
B. Heart failure
C. **Lung cancer**
D. Pulmonary embolism
E. Tuberculosis

### Example question with ethnicity

A 62 year old Indian man has 2 months of increasing shortness of breath and chest pain. He is now unable to lie flat. For the past 2 weeks, he has also had a productive cough which was flecked with blood on two occasions. He had a myocardial infarction 6 months ago, at which point he stopped smoking. His temperature is 37.1 °C, BP is 126/66 mmHg, respiratory rate is 24 breaths per minute, and oxygen saturation is 93% breathing air.

Investigations:

Chest X-ray:

moderate right sided pleural effusion

Pleural aspirate protein content 56 g/L

Which is the most likely underlying diagnosis?

A. Bacterial pneumonia
B. Heart failure
C. **Lung cancer**
D. Pulmonary embolism
E. Tuberculosis
